# Unraveling the viral dark matter through viral metagenomics

**DOI:** 10.3389/fimmu.2022.1005107

**Published:** 2022-09-16

**Authors:** Tasha M. Santiago-Rodriguez, Emily B. Hollister

**Affiliations:** Diversigen, Inc., Houston, TX, United States

**Keywords:** crAssphage, dark matter, microbiome, phage, virome, virus discovery

## Abstract

Viruses are part of the microbiome and have essential roles in immunology, evolution, biogeochemical cycles, health, and disease progression. Viruses influence a wide variety of systems and processes, and the continued discovery of novel viruses is anticipated to reveal new mechanisms influencing the biology of diverse environments. While the identity and roles of viruses continue to be discovered and understood through viral metagenomics, most of the sequences in virome datasets cannot be attributed to known viruses or may be only distantly related to species already described in public sequence databases, at best. Such viruses are known as the viral dark matter. Ongoing discoveries from the viral dark matter have provided insights into novel viruses from a variety of environments, as well as their potential in immunological processes, virus evolution, health, disease, therapeutics, and surveillance. Increased understanding of the viral dark matter will continue with a combination of cultivation, microscopy, sequencing, and bioinformatic efforts, which are discussed in the present review.

## Introduction

Viruses comprise the most abundant entities on Earth with an estimated 10^31^ particles (1). Viruses can be found wherever a potential host cell is present, and their numbers vary depending on the environment. For instance, an average of 10^7^ virus-like particles (VLPs) have been identified in a milliliter (mL) of sea water and urine (1, 2), while an average of 10^7^, 10^8^, and 10^8^ to 10^9^ VLPs have been identified in one mL of saliva (3), one gram of stool (4), and one gram of soil (5)(with new viruses increasingly being identified in soil) (6), respectively. Viruses are obligate intracellular parasites, with single-stranded (ss) or double-stranded (ds) DNA or RNA genomes (7), that replicate through a series of steps generally involving attachment to host surface receptors followed by replication and host cell lysis. Persistence of viruses may also depend on host cell availability and physiology (8), and certain viruses can remain dormant until conditions are favorable for replication and host cell lysis (9). Viruses are extremely diverse and include endogenous retroviruses, those infecting human, plant, or other animal and small eukaryotic cells, as well as bacterial viruses (i.e., phage) (10). While viruses are considered part of the microbiome, the term virome refers specifically to the collection of viruses present in a sample or community (11).

**Figure 1:**
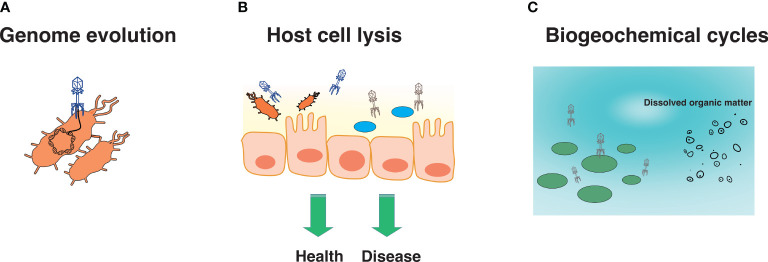
Several roles of viruses. Viruses are known to be involved in genome evolution **(A)**, host cell lysis, which may promote health or disease **(B)**, and biogeochemical cycles **(C)**.

Viruses are important and have been implicated in diverse processes ranging from evolution and immunity to biogeochemical cycles. Eukaryotic viruses may be directly or indirectly involved in the evolution of eukaryotic hosts. For instance, human and animal species contain considerable genetic diversity in their resistance against viral diseases, as in the case against certain retroviruses (12). While we often associate viruses with disease, most viruses are not directly pathogenic (13). Indeed, most of the virome is composed of phage, which may be neutral in their effects on the bacterial host, and be directly or indirectly involved in various processes. For instance, phage may impact bacterial evolution by altering genome composition through transduction (**Figure 1A**) (14). Transduction may also result in the acquisition of genes that may offer evolutionary advantages to the bacterial host (15), such as virulence factors and antibiotic-resistance genes, which in turn can turn out to be detrimental for human and animal health (16). Phage-encoded virulence factors, particularly those that facilitate adhesion in *Escherichia coli, Pseudomonas aeruginosa, Streptococcus mitis* and *Vibrio cholerae*, as well as invasion in *Salmonella enterica* and *Staphylococcus aureus*, may be associated with disease (17, 18). Similarly, certain phage are known to mediate the transfer of antibiotic resistance genes in *E. coli* strains including those conferring resistance to beta-lactams, tetracycline, ampicillin and kanamycin (19). Phage may also contribute to health or disease indirectly (discussed in this review) by altering the composition of specific bacterial communities (**Figure 1B**), and/or acting as a “second immune system” by lysing invading bacterial pathogens (20). In the environment, phage influence biogeochemical cycles by lysing bacteria and archaea, which then become dissolved organic matter used by heterotrophic bacteria. This, in turn, increases available nutrients, respiration, and CO_2_ production (**Figure 1C**) (21). Clearly, viruses influence a wide variety of systems and processes, and the continued discovery of novel viruses is anticipated to reveal new mechanisms influencing the biology and ecology of diverse environments.

## Virus discovery prior the metagenomics era

Virus discovery has been essential to understanding the emergence and re-emergence of viral pathogens, discovery, and characterization of non-pathogenic viruses, establishing model systems to study replication and infection mechanisms (e.g., T4 phage), and defining mechanisms that underpin immunological, evolutionary, and environmental processes. Prior the metagenomics era, viruses were typically discovered and understood using a variety of techniques aimed at deciphering replication and infection mechanisms, morphology, and genetic composition. In the following section, culture techniques, electron microscopy (EM), and molecular methods used for virus discovery are described and summarized (**Table 1** **)**. Sequencing techniques, described later in this review, are also presented in **Table 1**.

**Table 1:** Methods used for virus discovery.

Method	Example of virus discovered	Advantages	Limitations
Culture (i.e., *in vitro* and *in vivo*)	Adenoviruses; Polioviruses	Isolation of a wide variety of viruses including unexpected viruses and those in mixed cultures	Technical expertise needed to read cytopathogenic effects; Specialized cell lines and bacterial strains may be required; May take days to obtain results; Hard-to-culture bacteria may limit phage discovery and propagation
Electron microscopy (EM)	Tobacco mosaic virus (TMV); Monkeypox viruses	Viral morphology can be determined and facilitate virus classification; Useful for viruses that cannot be cultured	Highly trained personnel; Expensive equipment; Limit to detect viruses that replicate in the mitochondria and those that lack capsids
Molecular (e.g., PCR and RT-PCR)	SARS-CoV; SARS-CoV-2; Endogenous viral elements (EVEs)	High sensitivity and specificity; Short turn-around time; Useful for viruses that cannot be cultured or are hard to culture	Expensive due to costs of instrumentation and reagents; Possibility of false negatives when a virus has mutated; *A priori* knowledge may be needed
Viral metagenomics	crAssphage; crAss-like phage; “Quimbyviridae” candidate family; “Flandersviridae” candidate family; “Gratiaviridae” candidate family; Mimivirus of Acanthamoeba polyphaga; Rendondoviruses; Corona-like viruses	No *a priori* knowledge needed of the viral communities; No culture of the sample is needed; Simultaneously identify and characterize viruses of different families	Technical expertise may be required; Computational power for data analysis; Results may be biased depending on nucleic acid extraction method, concentration, nucleic acid amplification methods, sequencing and data analysis; Large number of sequences may not share homology to known viruses

This table highlights examples of viruses discovered using culture methods, electron microscopy (EM), molecular methods, and viral metagenomics. Advantages and limitations for each method are also described.

### Cell lines and bacterial strains

Culture methods involving cell lines (eukaryotic viruses) and bacterial strains (phage) have long been the gold standard for the discovery and characterization of viruses. Both *in vitro* and *in vivo* culture conditions have been used to identify and isolate a wide variety of viruses, including those that are in mixed culture (**Table 1**). Human and non-human cell lines have been used to identify viruses stemming from specific disease phenotypes (22). Interestingly, the initial discovery of some viruses did not involve the use of well-characterized cell lines; rather they were discovered by observing cytopathogenic effects in specific cells and tissues. This was the case with adenoviruses, first discovered in the 1950s, when it was noticed that an unknown agent was responsible for the cytopathogenicity of tissues originating from tonsils and adenoids (23). Similarly, poliovirus was originally discovered by the *in vivo* culture of the virus (which was unknown to be a virus at the time) in several different animals, including monkeys. It was then noticed that the virus caused the same effects to the monkey as those originally observed in humans (24).

Notably, potential drawbacks of *in vitro* or *in vivo* culture of viruses are the time and specific conditions required (**Table 1**). Viruses are not always straightforward to culture and may require technique optimization or the application of additional techniques for their identification and characterization. Hepatitis C (also known as non-A non-B hepatitis) is a prime example of this among eukaryotic viruses (25). Similarly, certain bacteriophage may be challenging to propagate. As with eukaryotic viruses, the identification and propagation of phage often relies on the use of relatively well-characterized cells (i.e., bacterial strains), which must be susceptible to infection and replication. Phage propagation also requires optimal media, temperature, and pH conditions to enable successful bacterial host growth, phage attachment, entry, replication, and bacterial lysis (26) (**Table 1**). Difficulty culturing phage and/or their bacterial hosts may limit their propagation, hampering our understanding of their morphological structure, and genome composition, as well as infection and replication mechanisms.

### Microscopy and molecular methods

Culture techniques are not always ideal for virus discovery and characterization. For this reason, other techniques, particularly EM, have been used in conjunction with culture for the discovery and characterization of viruses. EM, specifically, possesses the advantage over culture-based methods in that organism-specific reagents are not required (**Table 1**). The first virus visualized using EM was the *Tobacco mosaic virus* (TMV) in the 1930s. EM has also enabled the confirmation of certain Monkeypox and other poxviruses (27). Despite its benefits, EM can be expensive, requires highly-trained personnel, and may limit viral identification to the family-level as only morphology information can be obtained (**Table 1**).

Culture methods and EM may be accompanied by molecular techniques for virus discovery and characterization. Virus discovery is also possible through molecular techniques alone (28). Molecular methods have shown to be highly sensitive and specific, provide results with relatively short turn-around times, and be very useful for the identification and characterization of difficult-to-culture viruses (**Table 1**). Molecular methods using consensus primers, specifically, have also been applied for virus discovery. For example, highly divergent clades of human immunodeficiency virus (HIV) (29), and the Severe Acute Respiratory Syndrome associated coronavirus (SARS-CoV) (30), have been identified using consensus primers. SARS-CoV, in particular, was discovered after a patient’s sample tested negative for influenza, parainfluenza, respiratory syncytial virus, adenovirus, and a variety of bacterial pathogens. Since an unknown agent was suspected, the patient’s nasopharyngeal aspirate was subjected to Reverse Transcription (RT)-PCR using consensus primers targeting the coronavirus pol gene, which revealed a compatible gene product consistent with a novel coronavirus (31). Similarly, a combination of primers targeting coronaviruses aided in the discovery of the agent responsible for the most recent pandemic caused by SARS-CoV-2 (32). Molecular methods in combination with single genome bioinformatics have also enabled the discovery of endogenous viral elements (EVEs), which include retroviruses, DNA viruses, or RNA viruses (33). EVEs are known to be part of eukaryotic genomes, and their divergent hosts shows that some EVEs date back to approximately 100 million years (33). Some of these EVEs include bornaviruses (ss(-)RNA) (34), flaviviruses (ss(+)RNA) (35), circoviruses (ssDNA) (36), and hepadnaviruses (dsDNA) (37), which have been identified in the genomes of mammals, insects, and birds. While molecular techniques are indeed valuable for the discovery and further characterization of viral agents sharing homology to known viruses, a degree of *a priori* knowledge is typically needed, and false negatives may result in cases when the virus has mutated (**Table 1**).

## Viral metagenomics enables virus discovery

**Figure 2:**
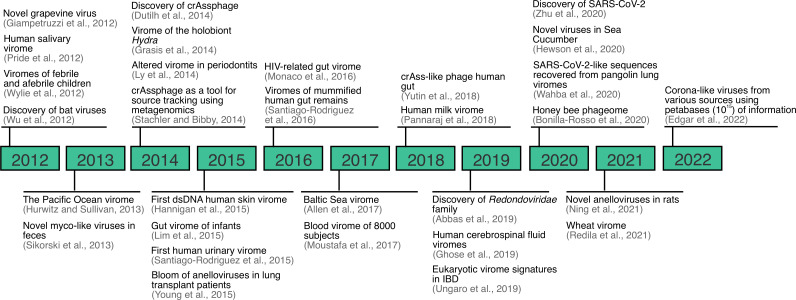
Overview of important viral metagenomic or virome studies landmarks between 2012-2022. Expanded from (28).

For the last 20 years, virus discovery has been facilitated by the application of metagenomic sequencing, and this continues to be an important tool for the discovery of viruses across a variety of sample types, environments and conditions (**Figure 2**) (2, 3, 28, 32, 38–62). Although metagenomic sequencing typically refers to DNA sequencing of mixed community of organisms, we refer to metagenomics here as the sequencing of DNA, RNA, or both. Viral metagenomics was applied for the first time in 2002 with the characterization of the virome of marine samples. The study found that over 65% of the sequences generated did not match available reference databases and that the identifiable fraction of the marine virome was mostly composed of dsDNA phage and algal viruses (63). Similarly, for the last decade, numerous discoveries have been made regarding the virome. For instance, saliva and other sample types, previously thought to be sterile (e.g., urine, blood, and cerebrospinal fluid), are now known to be home to robust communities of viruses that are not necessarily implicated with disease (2, 3, 51, 56). Another example includes crAssphage, which was discovered using a toolbox of bioinformatic methods aiming to characterize a DNA sequence shared across human stool samples (43). Finally, similar approaches have been applied to discover viral families from unexpected sources, as in the case of the discovery of the *Redondoviridae* family (55), and more recently, corona-like viruses from petabases (10^15^) of information (64).

Viral metagenomics provides advantages compared to culture, EM, and molecular-based methods in that no culture is required, no *a priori* knowledge of the viral communities in a sample type is necessarily needed, and diverse viral families can be characterized simultaneously (**Table 1**). Virus discovery from metagenomic data presents its own challenges, however, including nucleic acid extraction, amplification, library preparation, data analysis, and their associated biases, as described previously (65). An additional challenge associated with viral metagenomic data analysis comes from the lack of similarity of most of the viral sequences to known viruses in reference databases. Viruses are constantly evolving, as are viral databases, highlighting the importance of maintaining updated and curated viral databases to support viral metagenomic classification. For example, in some cases up to 99% of the putative viral sequences in a sample cannot be classified taxonomically due to the high degree of sequence divergence with known viruses (66). While this represents an opportunity for virus discovery, there are several criteria that should ideally be met for a putative virus to be considered novel and which may also be dependent on the bioinformatic methods used. For instance, the criteria for crAssphage to be considered a novel virus included, but were not limited to sharing a low sequence identity to known viruses in databases, and a high identity to putative or uncharacterized sequences originating from the same genome, an average sequence length (bp) of known viruses, ability to predict a putative bacterial host from the assembled genome (in case a phage is being suspected), and the ability to predict Open Reading Frames (ORFs) sharing homology to known viral proteins (43). Similar criteria have been used for the discovery of RNA viruses. In the case of SARS-CoV-2, for instance, the low homology (< 90%) to known viruses within betacoronaviruses was one of the main criteria to support this as a novel virus (67). Given that many constituents of viral dark matter are distantly related (at best) to available reference genomes, additional computational and cultivation efforts may be required to fully characterize a novel virus (68). In the following sections, examples of discoveries made from viral dark matter, and the techniques applied in these efforts, are discussed.

### dsDNA viruses: Lessons from crAssphage and other phage

Virus discovery through metagenomic sequencing is not trivial and typically requires the use of a variety of bioinformatic techniques including, but not limited to, *in silico* host sequence removal, read assembly, binning, alignment, co-occurrence assessment, phylogenetic characterization, and CRISPR analysis (**Table 2**). A bioinformatics framework incorporating each of these steps led to the discovery of crAssphage from human gut metagenomes and our understanding of *Prevotella* and *Bacteroides* spp. as its putative hosts (43, 77). As mentioned, this pioneering work identified a novel phage that we now know to be significantly distributed across the human gut (70), and predominant in the gut of industrialized cultures (78). The discovery of crAssphage also resulted in subsequent studies that enabled the discovery of crAss-like bacteriophage in human gut metagenomes by utilizing a similar suite of bioinformatic techniques, including searching for reads that did not align to nucleotide or protein reference databases, comparison with crAssphage proteins, and the prediction of open reading frames (**Table 2**) (69).

**Table 2:** Summary of bioinformatic tools used for virus discovery.

Virus discovered	Genetic material	Bioinformatics framework/[Other techniques]	Source/Origin	Reference(s)
crAssphage	dsDNA	Read assembly; Binning; Blastn; Re-assembly; Co-occurrence analysis; Open Reading Frame (ORF) prediction; CRISPR analysis	Human gut	(43)
crAss-like phage	dsDNA	psi-blast against non-redundant (nr) database; psi-blast of crAssphage protein candidates; Tblastn major capsid protein and other conserved proteins; Open Reading Frame (ORF) prediction; Phylogenetic analyses/ [Culture using a panel of bacteria; Sequencing of supernatant; Microscopy]	Human gut	(53, 69, 70)
Candidate families “Quimbyviridae”, “Flandersviridae”, “Gratiaviridae”	dsDNA	Protein predictions from downloaded assembled metagenomes; Hidden Markov Models; Phylogenetic analyses; CRISPR	Human gut	(71)
Various eukaryotic viruses	ssDNA	Read assembly; Blastn; Blastx; Blastp; Open Reading Frame (ORF) prediction; Protein structure predictions; Neural network analysis; Phylogenetic analyses/ [Gene expression; Microscopy]	Human skin; Human tissue	(72)
Giant viruses	dsDNA	Read assembly; Binning; Quality check of the bins to ensure no contamination	Water; Soil; Animals; Humans	(73)
SARS-CoV-2	ssRNA	Meta-transcriptomics/ [RT-PCR pancoronavirus primers]	Human respiratory tract	(32)
Various eukaryotic viruses	ssRNA; dsRNA	Read assembly; Blastx	Insecta; Crustacea; Myriapoda; Chelicerata; Nematoda; Annelida; Sipuncula; Mollusca; Platyhelminthes; Cnidaria; Echinodermata; Tunicata	(74)
Redondoviruses	scDNA	Read assembly; Open Reading Frame (ORF) prediction; Search for prokaryotic ribosomal binding sites; Phylogenetic analyses	Human respiratory tract	(55)
Corona-like virus	ssRNA	Read mapping; Read assembly; Palmprints	Various	(64)
Reoviruses, Flaviviruses, Permutotetraviruses, Nodaviruses, Negeviruses, Bunyaviruses, among others	dsRNA, ssRNA	Meta-transcriptomics; small RNA sequencing; Sanger sequencing; Phylogenetic analyses	*Drosophila;* mosquitoes; sandflies	(75, 76)

Other techniques applied for confirmation including culture and microscopy are also included in brackets.

Subsequent studies have since classified crAss-like phage sequences into several proposed subfamilies depending on their genome composition and predicted putative host, which involved the use of microscopy and culture-based methods. Microscopy analysis showed that most crAss-like phage possess morphologies (i.e., short, non-contractile tails) typical of the *Podoviridae* family (53). Additional studies identified a variety of gut bacteria, including *Agathobacter, Anaerostipes*, several *Bacteroides* (including *Bacteroides intestinalis*), *Blautia*, *Clostridium, Collinsella, Enterococcus*, and *Faecalibacterium* spp., as potential putative hosts for crAssphage and crAss-like phage; however, *B. intestinalis* was eventually confirmed to be crAssphage bacterial host based on sequencing gut filtrates and each bacterial host (79). Discovery, isolation, and characterization of crAssphage and crAss-like phage demonstrate the challenges of expanding currently available viral genomic databases with reliable taxonomic classification. For this reason, it is anticipated that frameworks combining culture, EM, viral metagenomic sequencing, and/or other molecular techniques will continue to be needed to facilitate bacteriophage discovery and characterization.

The approaches used in the discovery of other viruses vary widely but share many similarities with those used in the discovery of crAssphage and crAss-like phage. For instance, a multilevel bioinformatic framework for virus taxonomic classification enabled the discovery of other phage infecting Bacteroidetes, as well as the discovery of potentially novel phage families (80). Briefly, this involved the search of predicted proteins with phage-specific Hidden Markov Models, phylogenetic analysis, phage genome assembly, and CRISPR analyses to identify potential bacterial hosts. This resulted in the discovery of > 3,700 phage genomes covering > 450 genera, and the characterization of the candidate families “Quimbyviridae*”*, “Flandersviridae” and “Gratiaviridae” (**Table 2**) (71). Each of the candidate phage families were unique in terms of their genome structure and composition. For instance, phage from the candidate “Gratiaviridae” family encode a HipA-family protein kinase and glycosyltransferase, which suggest a role in host cell wall modification to prevent superinfection by other phage (80). This study is another example of a multi-level bioinformatic framework applied for phage discovery and characterization.

### Encountering eukaryotic DNA viruses in unexpected places

Eukaryotic viruses include viruses that infect humans, non-human primates, plants, fungi, insects, and other eukaryotes. Eukaryotic viruses are diverse in genome structure and composition, morphology, and replication and infection mechanisms; yet, phage are more frequently described in DNA viral dark matter studies than eukaryotic viruses. While eukaryotic viruses are often studied as pathological agents, not all eukaryotic viruses result in disease progression. Indeed, eukaryotic DNA viruses have been identified in samples collected from subjects with no apparent pathologies or history of related comorbidities. This was the case in the characterization of the human urine virome in subjects with and without urinary tract infections, where most of the identifiable reads corresponded to phage and samples from 19 of the 20 study subjects carried reads matching human papillomaviruses (HPVs) (2). These were not HPVs typically associated with urogenital samples, but rather were HPVs that have previously been identified in other sample types (2). This study is intriguing as it demonstrates that, while some eukaryotic viruses may not strictly fit the definition of viral dark matter as they have been previously identified, they can occur in unexpected places.

Similarly, numerous eukaryotic viruses have been identified as part of the DNA virome of humans and other animals but are endogenous to other sample types. For instance, pathogenic eukaryotic DNA viruses known to infect plants and insects have been identified as part of the bat gut virome since plants and insects are part of their diets (81). Specifically, ssDNA viruses, including animal viruses from the *Parvoviridae* and *Circoviridae* families, as well as plant viruses from the *Geminiviridae* family often dominate the DNA viral fraction of bat guano, and the proteins encoded by these viruses often exhibit less than 60% amino acid identity to known viral sequence proteins, suggesting the presence of numerous novel viral species in bats (81). These data highlight the importance of characterizing plant and other animal viruses as these may also provide insights into the virome composition of other samples and environments.

As with phage, there are instances when eukaryotic DNA viruses do not share homology with known sequences, highlighting the importance of searching both the DNA and protein space to enable virus discovery. For instance, ssDNA viral genes from human skin swabs and tissue samples were identified by focusing on sequences that lacked protein structural predictions (72). Leveraging assembly of these unannotated reads in combination with nucleotide, translated, and protein BLAST searches and artificial neural networks, led to the identification of ten novel genome groups containing at least one protein cluster predicted to encode virion structural proteins. A subset of the predicted capsid proteins were then expressed in human-derived 293TT cells and/or in *E. coli*, and EM results showed that several of the predicted capsid proteins formed rough spherical particles (**Table 2**) (72). These results are intriguing as they suggest that a suite of bioinformatic tools can positively predict viral sequences that can then be expressed, aiding in virus discovery.

Another intriguing example of DNA viruses infecting eukaryotic cells is that of giant viruses. Giant viruses are defined as viruses with genomes harboring over 500 protein-encoding genes, average genome sizes of over 1.02Mb, and capsids of 370 to 600 nm in diameter. Giant viruses were officially described in 2003 with the discovery of a virus infecting *Acanthamoeba polyphaga*, an amoeba, using microscopy-based methods (82). This *A. polyphaga* mimivirus was originally thought to be an intracellular bacterium due to its large structural size (83, 84). Since then, giant viruses have been discovered from various sample types including water, soil, and animals using culture-based methods (83), and more recently, they have been identified in viral dark matter using metagenomics (73). Using a combination of metagenomic assembly, genome binning, and quality assessment to ensure no contamination, it has been shown that giant viruses possess a complex machinery that is not usually found in viruses. Specifically, certain giant viruses have been shown to encode the components necessary for glycolysis and the TCA cycle (73). Other techniques, including a combination of sorting DNA-stained particles, coupled with whole genome amplification and sequencing have been applied for the discovery of giant viruses from soil samples (85). These results demonstrate the range and combination of techniques that can be applied for the discovery of giant viruses in various sample types.

### Meta-transcriptomics as a tool for eukaryotic RNA virus discovery

RNA viruses comprise most of the diversity of viruses infecting eukaryotic cells and have been historically discovered and characterized using culture and/or molecular-based methods (86, 87). RNA viruses, however, are not as well characterized as eukaryotic DNA viruses for several reasons (88). For instance, their small genome sizes make RNA viruses harder to detect in metagenomic data compared to DNA viruses, metagenomic sequencing methods targeting DNA tend to be better developed than those for RNA, and reference databases are typically biased towards DNA viruses and pathogens of economic importance. Each of these factors, and all of them in combination, makes RNA virus discovery more complex. High-throughput sequencing of RNA molecules, or meta-transcriptomics requires additional steps beyond a typical DNA-based metagenomic workflow in order to obtain reliable results. These include the synthesis of complementary DNA (cDNA) from messenger RNA (mRNA) and ribosomal RNA (rRNA) depletion.

RNA viruses have historically been of interest due to their potential to drive disease, epidemics, and pandemics, with SARS-CoV-2 being a recent example. Meta-transcriptomics, in combination with PCR techniques using pancoronavirus primers, enabled the discovery of SARS-CoV-2 (67). Meta-transcriptomics, however, is also a useful tool for the detection of non-pathogenic viruses. An example of this was the analysis of sequence data from 220 host species representing nine invertebrate phyla that led to the identification of >1,400 novel RNA viruses (74). In order to detect highly divergent viruses among these samples, the authors performed a combination of read assembly, domain-based blast against the Conserved Domain Database (CDD) for hits to RNA dependent RNA polymerases (RNA_dep_RNAP), and blastx searches of the putative viral assembled reads against the non-redundant (nr) protein database (**Table 2**), all while trying to balance discovery versus limiting false positive results (74). Several ssRNA, dsRNA, and dsDNA viruses were identified and discovered from the mentioned meta-transcriptomics datasets. As with the discovery of other viruses, this study is another example of the diversity of bioinformatic methods that can be used in conjunction with meta-transcriptomics to enable RNA virus discovery.

RNA virus discovery has also been enabled, in part, through small RNA sequencing. Particularly, small RNA sequencing has unraveled the role of RNA interference (RNAi) as an antiviral protection in insects, such as *Drosophila* spp. (75). Viruses in *Drosophila* spp. have been discovered historically using classical methods, which have enabled the discovery of viruses such Drosophila C Virus (DCV) (89), and Drosophila X Virus (DXV) (90). Further, transcriptomic analyses in *Drosophila melanogaster* revealed the presence of Nora virus (91). However, as with many environments, viral diversity in insects, including *Drosophila*, remains largely unexplored, but studies thus far have suggested an ongoing antiviral immune response in insects. Specifically, it has been suggested that the presence of RNAi in *Drosophila* spp. is characteristic of an antiviral response, and that these sequences may represent active viral infections. By using meta-transcriptomics and small RNA sequencing, sequence assembly, and targeted Sanger sequencing to improve completeness, over 20 partial viral genomes that comprised > 3.0% of all the sequences have been identified. The putative viruses identified included, but were not limited to Reoviruses, Flaviviruses, Permutotetraviruses, Nodaviruses, Negeviruses, and Bunyaviruses. Further analyses were not able to place the newly identified viruses within a phylogeny of known viruses, confirming their novelty (75). Similar approaches have been employed for sandflies and mosquitoes, showing six novel viruses belonging to viral families known to be pathogenic to mammals (e.g. *Bunyaviridae* and *Reoviridae* families) (76).

### Reducing the viral dark matter through artificial intelligence methods

As described in the previous sections, virus discovery from the dark matter often relies upon using sequence alignment methods against reference viral genes and genomes, as well as k-mer-based methods, which are used to predict a putative virus based on genomic and sequence signatures. Examples of several bioinformatic tools, as well as advantages and disadvantages for several of these sequence- and k-mer-based methods have previously been described (10). Other approaches, particularly Artificial Intelligence (AI), have also been applied in virus discovery from the viral dark matter. Specifically, deep learning, which uses deep artificial neural networks to ‘learn’ features from a given input and predict the output, have shown to be valuable in virus discovery from metagenomic datasets. Recent tools such as DeepVirFinder (92), and VIBRANT (93), utilize deep learning to predict viruses with success and higher accuracy compared to other methods.

## Viral dark matter in health and disease, therapeutic solutions and surveillance

**Figure 3:**
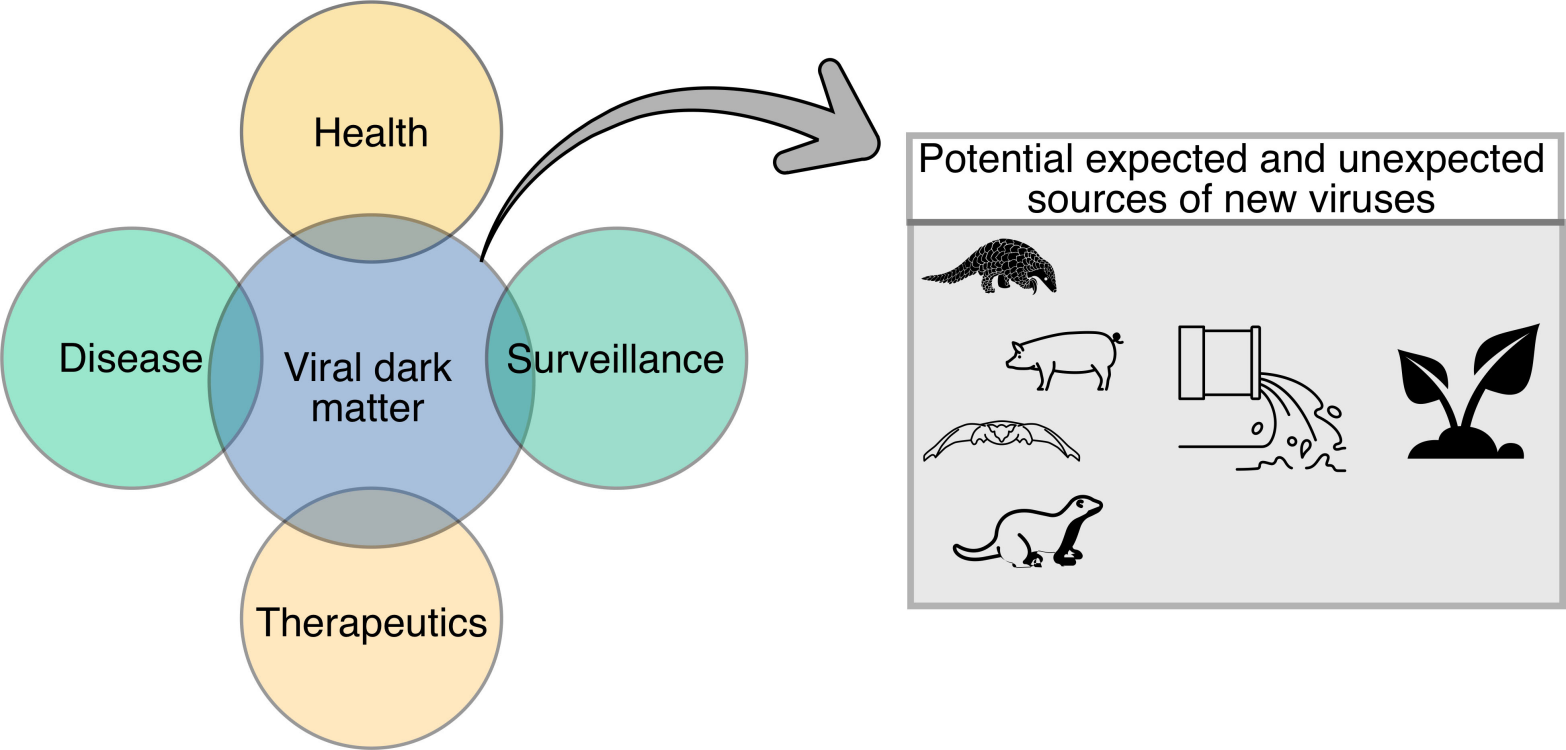
Diagram demonstrating potential applications of the viral dark matter in health, disease, therapeutics, and surveillance. Potential expected and unexpected sources of new viruses (i.e., animal, and environmental) that could contribute to the viral dark matter are also shown.

Viruses can be directly or indirectly associated with health and disease; yet, there is much to learn still about the contribution of the viral dark matter in health, disease, therapeutics, and surveillance efforts (**Figure 3**). The following section discusses known and unknown viruses in association with disease, including inflammatory bowel disease (IBD) and periodontitis, as well as the potential of the viral dark matter in therapeutics, particularly phage therapy, and surveillance.

### Inflammatory bowel disease

IBD is a chronic disorder of the intestinal tract that can result in periods of flare and remission. IBD, which includes Crohn’s disease (CD) and ulcerative colitis (UC), has an unknown etiology; yet, IBD appears to be multifactorial in nature and has been repeatedly associated with alterations of the human gut microbiome and more recently, the virome (94–96). Pioneer virome analyses of patients with IBD have found an increased viral richness and a decrease in bacterial diversity relative to control subjects, with the *Caudovirales* being the predominant viral order (94). Subsequent virome analyses of the same dataset focused on replication cycles (lytic vs lysogenic), as well as CRISPR analyses to determine potential bacterial host. The study identified an increased abundance of viruses belonging to the *Siphoviridae* and *Myoviridae* families, specifically those with lysogenic lifestyles (97). This study also found that a healthy gut virome is dominated by lytic phage, suggesting that these may be involved in lysing bacteria associated with disease progression (97). Notably, only 14% of the sequences were associated with known viruses in the original study, suggesting that that viral dark matter may be relevant to IBD onset and progression. These results also illustrate the need to expand viral DNA and RNA databases, which in turn can help elucidate the identity of uncharacterized viruses that are associated with disease.

### Periodontitis

The human oral cavity is the second most characterized body site after the human gut and is known to possess various biogeographical and ecological niches such as saliva, and subgingival and supragingival plaque. The human oral cavity is home to over 1,000 bacterial species including *Streptococcus* spp.*, Lactobacillus* spp. and *Veillonella* spp. (98, 99). Dysbiosis in the oral cavity can result in inflammation and the development of periodontal diseases, which can affect over 30% of the adult population. Historically, periodontitis has been associated with the ‘red complex’ which includes *Porphyromonas gingivalis, Treponema denticola* and *Tannerella forsythia* (100). However, periodontitis is likely multifactorial, having both immunological and microbial components (100).

The oral cavity also harbors viruses, many of which are associated with maintaining health or promoting disease (101). Interestingly, the oral cavity is characterized by having robust phage communities from the *Siphoviridae, Myoviridae* and *Podoviridae* families, each of which have been associated with health and periodontal disease (3). Specifically, myoviruses in supragingival plaque are predominant in subjects with periodontitis, whereas siphoviruses are predominant in the supragingival plaque of subjects without periodontal disease (45). Interestingly, the opposite has been noted in saliva samples, with myoviruses being predominant in subjects without periodontitis. Viral host taxonomy varies by sample type in subjects with periodontitis. For example, viruses infecting Firmicutes tend to be more abundant in saliva, while viruses infecting Actinobacteria tend to be more abundant in supragingival plaque. Phage infecting Bacteroidetes are also more predominant in individuals without periodontal disease (45). The proportion of identifiable viral reads in the oral cavity also tends to be low, but it has been reported to be as great as 40% in some cases. Given that well over half of the reads cannot be attributed to a specific viral source suggests that viral dark matter comprises most of the oral virome (45) and that the identity of most viruses in the oral cavity and their potential role(s) in health and disease remain to be elucidated.

Specific members of the viral dark matter have been associated with periodontal disease. A recent study showed the association of novel respiratory eukaryotic viruses with periodontal disease (55). Metagenomic sequencing of lung viromes, subsequent *de novo* read assembly, ORF prediction, prokaryotic ribosomal binding site searches, and phylogenetic analysis resulted in the discovery of a small circular DNA (scDNA) virus from a family named *Redondoviridae* (**Table 2**) (55). Further studies demonstrated that these were neither laboratory contaminants, nor phage. Further analysis of metagenomes from subjects with periodontal disease found that redondoviruses were predominant in these datasets (55). These data are intriguing as it shows an association between inflammation and the respiratory viral dark matter.

### Therapeutic solutions: Lessons from phage therapy

As described, phage can make up a large fraction of the viral dark matter in some environments. This universality of phage, their host-specificity, and their ability to lyse bacterial hosts make them ideal candidates to treat infections caused by antibiotic-resistant bacteria (ARB). The application of phage in this way, also known as phage therapy, has shown renewed interest in recent years as a way to treat ARB infections (102). Initial disinterest in phage therapy arose from mistakes made during early trials, along with the discovery of antibiotics, which made treatment relatively straightforward. However, phage therapy has again proven to be effective against infection caused by various bacteria including, but not limited to *Pseudomonas aeruginosa* (102, 103), *Clostridium difficile* (104), and *Enterococcus faecalis* (105). Single bacteriophage and phage cocktails are readily available and can be used to target certain ARB.

Phage therapy can also become personalized when available phage and phage cocktails do not efficiently lyse target ARB. Screening for ideal phage candidates to be used in phage therapy can be time and labor intensive. The search for phage that could efficiently lyse target ARB can involve screening samples, such as sewage (as it is an ideal sample type to find human-associated viruses) (106). Once a candidate phage is found, it should be further characterized to understand host range, as ideal phage should be specific to the bacterial strain of interest (106). In some cases, the candidate phage may be known, but in other cases, suitable phage can be unknown and be part of the viral dark matter; therefore, genome sequencing and characterization should be performed to understand genome composition and confirm potential bacterial host. Genome characterization will also ensure that the phage is strictly lytic and that complete or near complete lysis of the bacteria causing infection will be accomplished. In the case of the candidate phage being lysogenic or temperate, genome engineering may be considered as an approach to produce suitable phage candidates for phage therapy against specific pathogens (107).

### Surveillance through viral metagenomics of the viral dark matter

Understanding the source and evolution of potential emerging and re-emerging pathogens is essential for surveillance efforts. This is when viral metagenomics becomes an important surveillance tool as it allows numerous viruses to be identified simultaneously from various sources and facilitates using one or several of the above-mentioned tools. Virus discovery from viral dark matter is increasingly recognized as an important aspect of surveillance efforts. For instance, while bats are recognized as a resource of novel coronaviruses and an important host for coronavirus evolution (108, 109), viral metagenomic approaches are identifying a variety of novel and unexpected hosts of corona-like viruses (110). Specifically, a recent study leveraging high-throughput compute infrastructure, RNA-dependent RNA polymerase sequences (RdRP), which are characteristic of RNA viruses lacking a DNA stage, and >3 million metagenomics, metatranscriptomics, and virome datasets identified over 130,000 novel RNA viruses, nine of which were novel coronaviruses (64). These novel RNA viruses represented approximately 0.1% of the total virome analyzed. The study also concentrated on corona-like viruses including Microhyla alphaletovirus 1 (MLeV) in the frog *Microhyla fissipes*, and Pacific salmon nidovirus (PsNV), identifying samples containing corona-like virus-aligned reads and/or k-mers and performing *de novo* assembly using coronaSPAdes (111). This resulted in 70 species-like operational taxonomic units (sOTUs), 44 of which were found to be described in public databases, and 17 corona-like virus sOTUs contained partial RdRP. The remaining nine sOTUs were identified as novel corona-like viruses, as they exhibit protein domains consistent with a corona-like viral genome (**Table 2**). This study has revolutionized virome and viral dark matter research in that it has introduced an approach for petabase (10^15^)-scale genomics, which can aid in reducing the viral dark matter and improve surveillance efforts of potential emergent viruses from unexpected sources.

## Conclusions, challenges, and future directions

For the last 20 years, viral metagenomics has shown to be a powerful tool for the discovery of viruses. Viral metagenomics continues to be essential in the characterization of known viruses in diverse sample types, and in association with health, disease, immune system, biogeochemical cycles, therapeutics, and surveillance. Moreover, identification of novel viruses from viral dark matter continues to be possible with viral metagenomics. Virus discovery through viral metagenomics, however, does not come without challenges. Many such challenges that are intrinsic to the technique including viral purification, nucleic acid extraction and amplification, library preparation, sequencing, and bioinformatics. Examples of the bioinformatic frameworks that could be applied for virus discovery from viral dark matter are highlighted in the present review and demonstrate that no universal framework applies to the discovery of all viruses, but are rather diverse and should be suited for the virus(es) of interest. Virus discovery from viral metagenomics data may also require the application of culture, molecular and/or EM techniques.

## Author contribution

TS-R original draft preparation. TS-R and EH: review and editing of manuscript draft. All authors contributed to the article and approved the submitted version.

## Conflict of interest

TS-R and EH are current employees of Diversigen, a subsidiary of OraSure Technologies and a microbiome services company.

## Publisher’s note

All claims expressed in this article are solely those of the authors and do not necessarily represent those of their affiliated organizations, or those of the publisher, the editors and the reviewers. Any product that may be evaluated in this article, or claim that may be made by its manufacturer, is not guaranteed or endorsed by the publisher.
